# Measuring Safety and Outcomes for the Use of Compassionate and Off-Label Therapies for Children, Adolescents, and Young Adults With Cancer in the SACHA-France Study

**DOI:** 10.1001/jamanetworkopen.2023.21568

**Published:** 2023-07-03

**Authors:** Pablo Berlanga, Lee Aymar Ndounga-Diakou, Isabelle Aerts, Nadège Corradini, Stéphane Ducassou, Marion Strullu, Emilie de Carli, Nicolas André, Natacha Entz-Werle, Sandra Raimbault, Marianne Roumy, Marjolaine Renouard, Gwenaelle Gueguen, Dominique Plantaz, Yves Reguerre, Morgane Cleirec, Arnaud Petit, Chloe Puiseux, Leslie Andry, Sébastien Klein, Damien Bodet, Justyna Kanold, Claire Briandet, Carine Halfon-Domenech, Brigitte Nelken, Christophe Piguet, Laure Saumet, Pascal Chastagner, Joy Benadiba, Frédéric Millot, Claire Pluchart, Pascale Schneider, Sandrine Thouvenin, Marion Gambart, Jill Serre, Samuel Abbou, Amaury Leruste, Hélena Cayzac, Virginie Gandemer, Salim Laghouati, Gilles Vassal

**Affiliations:** 1 Department of Pediatric and Adolescent Oncology, Gustave Roussy Cancer Campus, Université Paris-Saclay, Villejuif, France; 2 Pharmacovigilance Unit, Clinical Research Direction, Gustave Roussy Cancer Campus, Université Paris-Saclay, Villejuif, France; 3 SIREDO Oncology Center (Care, Innovation and Research for Children and AYA with Cancer), Institut Curie, PSL Research University, Paris, France; 4 Paediatric Haematology-Oncology Institut, of Centre Léon Bérard, Lyon, France; 5 Paediatric Haematology-Oncology Department, Centre Hospitalier Universitaire (CHU), Bordeaux, France; 6 Paediatric Immuno-Haematology Department, Hôpital Robert-Debré AP-HP, Paris, France; 7 Department of Pediatric Oncology, University Hospital, Angers, France; 8 Department of Pediatric Hematology & Oncology, Hôpital de La Timone, AP-HM, Marseille, France; 9 Pediatric Onco-Hematology Unit, University Hospital of Strasbourg, Strasbourg, France; 10 Department of Pediatric Oncology, Oscar Lambret Cancer Center, Lille, France; 11 Department of Pediatric Onco-Immuno-Hematology, Grenoble Alpes University Hospital, Grenoble, France; 12 Pediatric Oncology and Hematology Unit, CHU Saint Denis de la Réunion, Bellepierre, France; 13 Pediatric Immuno-Hemato-Oncology Unit, CHU Nantes, Nantes, France; 14 Department of Pediatric Hematology and Oncology, Hôpital Armand Trousseau, Paris, France; 15 Department of Pediatric Hemato-Oncology, University Hospital of Rennes, Rennes, France; 16 Department of Pediatric Oncology, CHU Amiens Picardie, Amiens, France; 17 Pediatric Oncology and Hematology, CHU Jean-Minjoz, Besançon, France; 18 Department of Pediatric Hematology and Oncology, University Hospital of Caen, Caen, France; 19 Department of Pediatric Hematology-Oncology, University Hospital of Clermont-Ferrand, Clermont-Ferrand, France; 20 Pediatric Oncology, CHU Dijon-Bourgogne, Dijon, France; 21 Department of Pediatric Hematology-Oncology, University Hospital of Lyon, Lyon, France; 22 Department of Pediatric Hematology-Oncology, Jeanne de Flandre Hospital, CHRU, Lille, France; 23 Pediatric Oncology, CHU Limoges, Limoges, France; 24 Department of Pediatric Hematology-Oncology, University Hospital of Montpellier, Montpellier, France; 25 Department of Pediatric Hematology-Oncology, Children’s Hospital of Brabois, Vandoeuvre Les Nancy, France; 26 Department of Hemato-Oncology Pediatric, Nice University Hospital, Nice, France; 27 Department of Paediatric Haematology and Oncology, Centre Hospitalo-Universitaire de Poitiers, Poitiers, France; 28 Department of Paediatric Haematology and Oncology, Centre Hospitalo-Universitaire de Reims, Reims, France; 29 Department of Pediatric Hematology and Oncology, Centre Hospitalo-Universitaire de Rouen, Rouen, France; 30 Department of Pediatric Hematology-Oncology, University Hospital St Etienne, St Etienne, France; 31 Department of Pediatric Oncology, Toulouse University Hospital, Toulouse, France; 32 Department of Pediatric Hematology-Oncology, CHRU de Tours, Tours, France

## Abstract

**Importance:**

Innovative anticancer therapies for children, adolescents, and young adults are regularly prescribed outside their marketing authorization or through compassionate use programs. However, no clinical data of these prescriptions is systematically collected.

**Objectives:**

To measure the feasibility of the collection of clinical safety and efficacy data of compassionate and off-label innovative anticancer therapies, with adequate pharmacovigilance declaration to inform further use and development of these medicines.

**Design, Setting, and Participants:**

This cohort study included patients treated at French pediatric oncology centers from March 2020 to June 2022. Eligible patients were aged 25 years or younger with pediatric malignant neoplasms (solid tumors, brain tumors, or hematological malignant neoplasms) or related conditions who received compassionate use or off-label innovative anticancer therapies. Follow up was conducted through August 10, 2022.

**Exposures:**

All patients treated in a French Society of Pediatric Oncology (SFCE) center.

**Main Outcomes and Measures:**

Collection of adverse drug reactions and anticancer activity attributable to the treatment.

**Results:**

A total of 366 patients were included, with a median age of 11.1 years (range, 0.2-24.6 years); 203 of 351 patients (58%) in the final analysis were male. Fifty-five different drugs were prescribed, half of patients (179 of 351 [51%]) were prescribed these drugs within a compassionate use program, mainly as single agents (74%) and based on a molecular alteration (65%). Main therapies were *MEK*/*BRAF* inhibitors followed by multi-targeted tyrosine kinase inhibitors. In 34% of patients at least a grade 2 clinical and/or grade 3 laboratory adverse drug reaction was reported, leading to delayed therapy and permanent discontinuation of the innovative therapy in 13% and 5% of patients, respectively. Objective responses were reported in 57 of 230 patients (25%) with solid tumors, brain tumors, and lymphomas. Early identification of exceptional responses supported the development of specific clinical trials for this population.

**Conclusions and Relevance:**

This cohort study of the SACHA-France (Secured Access to Innovative Medicines for Children with Cancer) suggested the feasibility of prospective multicenter clinical safety and activity data collection for compassionate and off-label new anticancer medicines. This study allowed adequate pharmacovigilance reporting and early identification of exceptional responses allowing further pediatric drug development within clinical trials; based on this experience, this study will be enlarged to the international level.

## Key Points

**Question:**

Is it feasible to collect prospective clinical safety and activity data for the use of compassionate or off-label innovative anticancer medicines at a multicenter, national level?

**Findings:**

This cohort study included information collected from 366 children, adolescents, and young adults treated with 55 compassionate or off-label innovative drugs as part of the SACHA (Securing Access to Innovative Therapies for Children, Adolescents, and Young Adults with Cancer Used Outside Clinical Trials)-France study. Pharmacovigilance monitoring confirmed significant adverse drug reactions in one-third of them; 25% of the patients achieved at least a partial response.

**Meaning:**

These results confirm the feasibility of this prospective real-word study.

## Teaser

This cohort study of children, adolescents, and young adults treated at pediatric oncology centers in France examines outcomes and safety measures for the use of off-label treatments for cancer.

## Introduction

Childhood and adolescent cancers comprise a heterogeneous group of rare diseases, mostly distinct to those diagnosed in adults. Survival of children and adolescents with cancer has considerably improved in recent decades, but cancer remains the leading cause of disease-related mortality in high-income countries.

Although better understanding of cancer biology has led to increased development of novel targeted therapies and immunotherapies in the last 2 decades, only few have been approved for the treatment of childhood cancers over the last 15 years. There are more anticancer medicines approved for the treatment of childhood cancers in the US than in the EU, with a median time of 1.9 years between pediatric anticancer drug authorization by the US Food and Drug Administration (FDA) and the authorization in the EU.^1^

In France, since January 2016 all pediatric and young adult patients with recurrent or refractory malignant neoplasms have access to tumor molecular profiling at the time of disease recurrence, first within the MAPPYACTS trial^2^ and, since 2020, within the French National Program, the 2025 France Genomic Medicine Initiative (PFMG2025) as part of health care. In order to increase the access to matched innovative therapies within the framework of early phase clinical trials the Innovative Therapies for Children with Cancer (ITCC) AcSé-ESMART platform trial (NCT02813135) was open in parallel in France and Europe in 2016 in addition to early phase trials running in the ITCC centers. However, the current clinical trials portfolio still remains insufficient and pediatric hematologist-oncologists regularly prescribe unauthorized drugs on compassionate use and/or innovative medicines outside their marketing authorization and clinical trials. Before 2020, no information on those treatments was collected in France. In order to collect clinical data of the administration of compassionate and off-label used innovative therapies, the French Society of Pediatric Oncology (SFCE) developed in 2020 the Secured Access to Innovative Medicines for Children with Cancer (SACHA)-France observational study (NCT04477681).

## Methods

### Study Design and Procedure

The SACHA-France study is a prospective, multicenter, national observational registry that collects clinical data of innovative therapies (targeted therapies, immunotherapy, or chemotherapy) administered to patients aged 25 years or younger at the time of prescription with pediatric malignant neoplasms (solid tumor or leukemia) or related conditions, provided through the French early access authorization and compassionate use programs or as an off-label anticancer medicine that has been first approved in adults in Europe after the implementation in 2017 of the EU Pediatric Regulation.^3^ Before inclusion in the SACHA-France study, interregional multidisciplinary discussion is mandatory to ensure that all patients receive the most adapted available treatment, preferably within clinical trials. This study is a Research Involving Human Subjects of category 3 (noninterventional studies) according to the French Jardé law, that request patient or their parents or legal representative’s nonopposition. This study has been reviewed and approved by the French ethics committee in compliance with the EU General Data Protection Regulation, which confirmed that informed consent was not needed. SACHA-France is open in all SFCE centers. We followed the Strengthening the Reporting of Observational Studies in Epidemiology (STROBE) reporting guideline.

### Adverse Drug Reactions Reporting

Adverse drug reactions (ADRs) attributable to the treatment under study are described according to the Common Terminology Criteria for Adverse Events (CTCAE) version 5. Only ADRs grade 2 or higher for clinical and/or 3 or above for laboratory are reported. All reported ADRs that lead to drug dose reduction, delay, or end of treatment as well as all unexpected ADRs and all serious ADRs (SADRs) are remotely monitored by the Pharmacovigilance Unit of Gustave Roussy.

### Outcome Assessments

Progression-free survival (PFS) and best response from the start of treatment to tumor relapse or progression is assessed by the patient’s treating physician following the standard response criteria for each tumor type. In case of reported objective tumor response (ORR), radiological reports at baseline or time of response are reviewed by the SACHA-France coordinating investigator to confirm the coherence of the reported response.

### Data Analysis

All cohorts of 10 patients or more with measurable or evaluable disease treated with the same drug for the same indication and reported at least 6-month follow-up as well as patients with observed antitumor activity previously not reported are reviewed by the SACHA France Steering Committee. In cases where no activity is observed or if there is a major safety concern (severe toxic effects or death), the steering committee recommends to all SFCE centers to no longer prescribe the medicine in that specific indication. If outstanding not previously reported activity results are observed, the steering committee recommends the development of an early phase clinical trial in that specific indication.

### Statistical Analysis

Categorical variables were described with the frequency and percentage of each category; continuous variables as median and range value.

## Results

From March 2020 to June 2022, 366 patients were included in 30 SFCE centers. Fifteen patients did not fulfill the inclusion criteria and were considered as screening failures (eTable 1 in Supplement 1). Thus, 351 patients were analyzed, with a median age at time of start of innovative therapy was 11.1 years (range, 0.2-24.6 years) (203 male [58%]). Of them, 33 patients were 18 years or older; 19 patients received more than 1 therapy. Compassionate or off-label therapies were administered mainly as single agent (258 patients [74%]) and were used based on a tumor molecular alteration in 220 of 341 of reported patients (65%). Prior to the compassionate or off-label therapy, patients had received a median of 2 prior lines of treatment (range, 0-9) with a median time of 2.0 years since initial diagnosis (range, 0-16.5 years). All patients were treated at the time of relapse or progression, except 17 patients (*NF1*-related plexiform neurofibroma treated by trametinib [10 patients]; *BRAF*-altered low grade glioma [LGG] treated by dabrafenib plus trametinib [4 patients]; *NTRK* fusion–positive sarcomas treated by larotrectinib [3 patients]). Main tumor types of 342 reported patients were central nervous system (CNS) tumors (164 patients [48%]), non-CNS solid tumors (122 patients [36%]), leukemias (36 patients [11%]), and lymphomas (20 patients [6%]) (Table 1).

**Table 1.** Characteristics of Patients Included in the SACHA-France Study

Diagnosis	Patients, No. (N = 342)	No. of patients with ≥2 therapies	Targeted molecular alteration (No. of patients)^a^	Innovative therapies (No. of patients)^a^
**CNS tumors (164 patients)**				
Low-grade glioma	95	3	*BRAF* variant (35); *BRAF* fusion–positive (28); *BRAF* duplication (2); *NF1* germ variant (15)	Trametinib (54); dabrafenib plus trametinib (36); selumetinib (2)
High-grade glioma	46	3	*H3K27M* variant (21); *BRAF* variant (7); *ACV1* variant (2)	ONC201 (24); Dabrafenib plus trametinib (5); trametinib (3); vandetanib (2)
Atypical teratoid rhabdoid tumor	7	NA	SMARCA/B deletion/variant (7)	Tazemetostat (7)
Ependymoma	2	1	NA	NA
Medulloblastoma	4	NA	*PTCH1* variant (3)	Vismodegib (3)
Other CNS tumors	10	4	*MET* fusion (3); ALK fusion (2)	Crizotinib (3)
**Non-CNS tumors (122 patients)**				
Osteosarcoma	24	1	NA	Regorafenib (14); cabozantinib (10)
Ewing sarcoma	17	NA	NA	Cabozantinib (9); regorafenib (5); pazopanib (2)
Plexiform neurofibroma	13	NA	*NF1* germ variant (12)	Trametinib (11); selumetinib (2)
Neuroblastoma	12	NA	*ALK* variant (5)	Lorlatinib (5); Dinutuximab beta/chemotherapy (4)
Desmoid tumor	6	NA	NA	Pazopanib (6)
Rhabdomyosarcoma	5	NA	NA	Pazopanib (3)
Thyroid carcinoma	5	NA	*RET* variant (2); *SMARCA/B* deletion/variant (2)	Tazemetostat (2)
Inflamatory myofibroblastic tumor	4	NA	*ALK* fusion (2)	Crizotinib (3)
Hepatoblastoma	3	1	NA	NA
Infantile fibrosarcoma	3	NA	*NTRK* fusion (3)	Larotrectinib (3)
Melanoma	3	NA	NA	Nivolumab (2)
Other soft tissue sarcoma	11	1	*NTRK* fusion (4)	Larotrectinib (3); pazopanib (3)
Other non-CNS tumors	15	1	*BRAF* variant (3); *SMARCA/B* deletion/variant (2)	Dabrafenib plus trametinib (2); pazopanib (2); tazemetostat (2); atezolizumab (2)
**Lymphoma (20 patients)**				
Hodgkin lymphoma	10	NA	NA	Brentuximab/nivolumab (6); brentuximab (4)
Non-Hodgkin lymphoma	10	NA	*ALK* fusion (7)	Alectinib (6); anti-CD20/CD3 (2)
**Leukemia (36 patients)**				
Acute myeloblastic leukemia	16	2	*ALK* fusion (2)	Venetoclax/azacitidine (9); venetoclax (3)
B-cell acute lymphoblastic leukemia	12	0	*BCL-ABL* variant (2)	Inotuzumab (6); ponatinib (3)
T-cell acute lymphoblastic leukemia	1	NA	NA	NA
Other leukemia	7	2	NA	NA

Abbreviations: CNS, central nervous system; NA, not applicable.

^a^ Minimum 2 patients.

### Innovative Therapies Characteristics

Fifty-five different drugs were registered, with 25 prescribed more than once (Figure; eTable 2 in Supplement 2). Half of all prescriptions (179 of 351 [51%]) were performed within a compassionate use program authorized by the French competent authority (French National Agency for the Safety of Medicines and Health Products [ANSM]).

**Figure.**
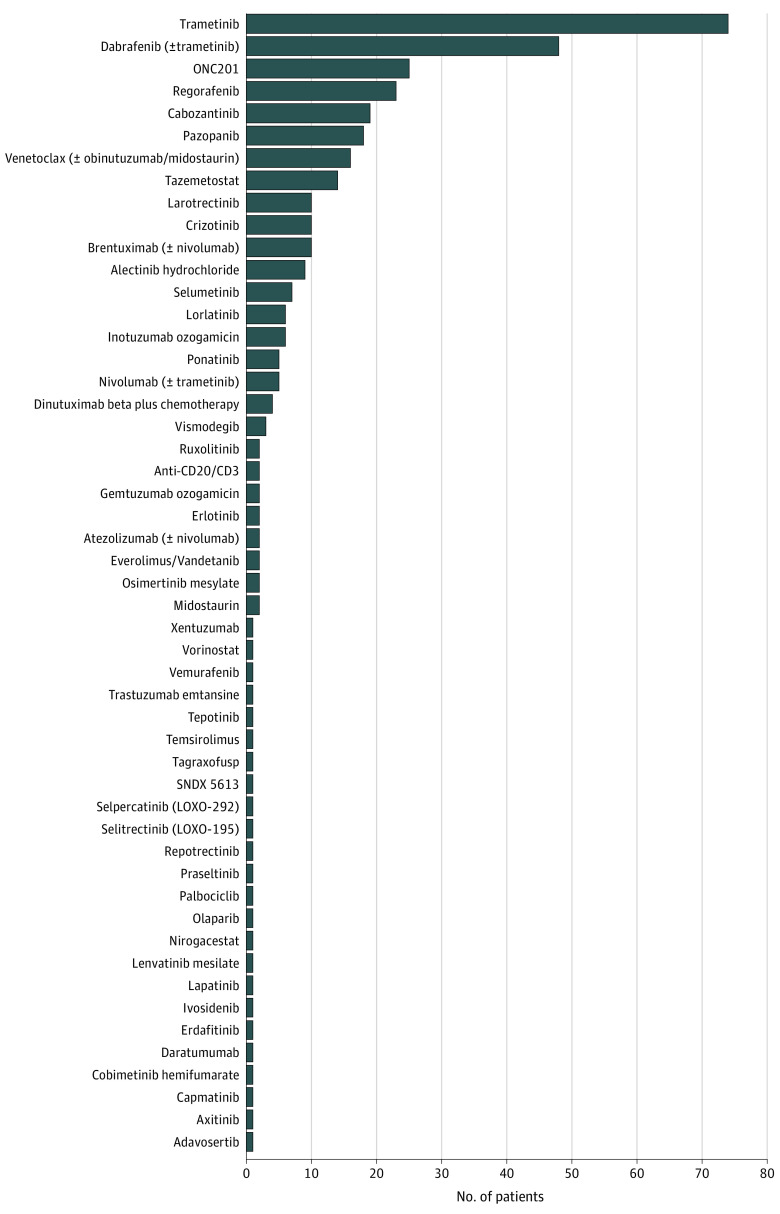
Drugs Included in the SACHA-France Study

Main innovative therapies prescribed in 10 patients or more were trametinib (74 patients), dabrafenib plus trametinib (44 patients), ONC201 (25 patients), regorafenib (23 patients), cabozantinib (19 patients), pazopanib (18 patients), tazemetostat (14 patients), venetoclax (12 patients), larotrectinib (10 patients), and crizotinib (10 patients). Main reported genetic alterations that led to a matched targeted therapy were alterations in *BRAF* (84 patients), *NF1* (30 patients), *H3K27M* (21 patients), *ALK* (20 patients), *SMARCA/B12* (10 patients), and *NTRK* (10 patients).

Information on follow-up was provided for 322 of 351 patients (91%). Treatment was stopped for 194 of 322 patients (60%). Main reasons for end of treatment were disease progression (124 patients [64%]), physician decision (38 patients [20%], mainly because treatment was given as consolidation or maintenance therapy, or patient treatment was bridging to another therapy), adverse drug reaction (17 patients [9%]), disease progression and adverse drug reaction (4 patients [2%]), and patient’s decision.^4^ None of the study participants were lost to follow-up.

### Adverse Drug Reactions Reported

With a date cut-off of the August 10, 2022, 284 CTCAE grade 2 to 4 ADRs related to 26 out of 55 prescribed innovative therapies were reported in 121 of 351 patients eligible for analysis (34%). More than a third of them (122 of 284 patients [43%]) were related to trametinib as a single agent.

Overall, 72 of 284 ADRs (25%) led to a delayed therapy in 45 patients, 38 of 284 (13%) to a drug reduced dose in 23 patients, and 29 of 284 (10%) to a discontinued treatment in 22 patients (related to 12 different therapies), including 5 patients with a temporarily interruption only. All nonserious ADRs reported are described in eTable 3 in Supplement 1.

A total 24 SADRs (8%) related to 12 therapies were reported in 17 patients (Table 2). Of them, 8 (33%) led to a delayed therapy (6 patients), 5 (21%) to a temporary stop (3 patients), and 8 (33%) to definitive therapy stop (6 patients), including a grade 4 life-threatening secondary malignant neoplasm (peripheral T cell lymphoma) in a patient younger than 5 years with an atypical teratoid/rhabdoid tumor (ATRT) previously treated by chemotherapy, surgery, and radiotherapy and with a long-lasting response to tazemetostat (14 months). All SADRs were reported to regional pharmacovigilance centers, which relayed them to the ANSM.

**Table 2.** Characteristics of SADRs

Characteristic	Patients, No. (%) (N = 351)				Innovative therapy of interest	Diagnosis	Action taken with the therapy of interest	Expectedness	Corrective treatment
	All grade	Grade 2	Grade 3	Grade 4					
No. of patients who received at least 1 dose of treatment with an SADR	17 (4.8)	2 (0.5)	14 (3.9)	3 (0.8)	NA	NA	NA	NA	NA
Gastrointestinal disorders									
Abdominal pain	1 (0.2)	0	1 (0.2)	0	Azacitidine; venetoclax	Acute myeloid leukemia	Temporary stop	Expected	Yes
Diarrhea	1 (0.2)	0	1 (0.2)	0	Dabrafenib mesylate; trametinib	Low grade glioma	Delayed	Expected	Yes
Macroglossia	1 (0.2)	0	1 (0.2)	0	Inotuzumab ozogamicin	B-cell lymphoblastic leukemia	Definitive stop	Unexpected	Yes
General disorders and administration site conditions									
Vomiting	1 (0.2)	0	1 (0.2)	0	Selumetinib	Low grade glioma	No action	Expected	Yes
Fever	2 (0.5)	1 (0.2)^a^	1 (0.2)	0	Dabrafenib mesylate; trametinibb	Optic nerve glioma; Low grade glioma	Delayed	Expected	No
Infections and infestations									
Acute pyelonephritis	2 (0.5)	1 (0.2)^a^	1 (0.2)	0	Dabrafenib mesylate; trametinibc	Low grade glioma	Delayed	Expected	Yes
Metabolism and nutrition disorders									
Hyponatremia	1 (0.2)	0	1 (0.2)	0	Trametinib	Low grade glioma	Delayed	Expected	Yes
Nervous system disorders									
Neuralgia	1 (0.2)	0	1 (0.2)	0	Lenvatinib mesilate	Brain stem glioma	Definitive stop	Unexpected	Yes
Psychiatric disorders									
Delirium	1 (0.2)	0	1 (0.2)	0	Alectinib hydrochloride (associated with antiepileptics)	Non-Hodgkin Lymphoma	Delayed	Unexpected	No
Respiratory, thoracic, and mediastinal disorders									
Pneumothorax	1 (0.2)	0	1 (0.2)	0	Regorafenib	Ewing sarcoma	Delayed	Unexpected	Yes
Skin and subcutaneous tissue disorders									
Rash maculo-papular	1 (0.2)	0	1 (0.2)	0	Regorafenib	Ependymoma	Definitive stop	Expected	Yes
Dry skin	1 (0.2)	0	1 (0.2)	0	Trametinib;	Low grade glioma	Dose reduced/temporarily stop	Expected	Yes
Dehiscenced surgical wounds	4 (1.1)	0	2 (0.5)	2 (0.5)	Regorafenibc	Osteosarcoma	Definitive stop	Expected	No
Benign, malignant, and unspecified neoplasms (including cysts and polyps)									
Treatment related secondary malignancy	1 (0.2)	0	0	1 (0.2)	Tazemetostat	Atypical teratoid/rhabdoid tumor	Definitive stop	Unexpected	Yes
Alanine aminotransferase increased	1 (0.2)	0	1 (0.2)	0	Crizotinibd	Acute myeloid leukemia	Definitive stop	Expected	No
Aspartate aminotransferase increased	1 (0.2)	0	1 (0.2)	0	Crizotinibd	Acute myeloid leukemia	Definitive stop	Expected	No
Vascular disorders									
Capillary leak syndrome	1 (0.2)	0	0	1 (0.2)	Tagraxofusp	Other leukemia	No action	Expected	Yes
Metabolism and nutrition disorders									
GGT increased	1 (0.2)	0	1 (0.2)	0	Crizotinibd	Acute myeloid leukemia	Definitive stop	Expected	Yes
Blood and lymphatic system disorders									
Febrile neutropenia	1 (0.2)	0	1 (0.2)	0	Dinutuximab beta (plus topotecan/cyclophosphamide)	Neuroblastoma	No action	Expected	Yes

Abbreviations: ADR, adverse drug reactions; GGT, gamma-glutamyltransferase; SADR, serious adverse drug reaction.

^a^ This ADR led to an hospitalization.

### Outcome of Patients

Of the 242 patients with solid tumors, brain tumors, or lymphomas with treatment started before December 31, 2021, 230 (95%) had measurable or evaluable disease at study entry and were evaluable for response assessment. Of them, 57 of 230 (25%) achieved a partial response or complete response as best response (47 and 10 patients, respectively), confirmed by remote monitoring of radiological reports at baseline or time of response. Most responses were related to drugs already approved by the European Medicines Agency or the FDA for adults or other indications (54 of 57 drugs) (eTable 4 in Supplement 2), although 3 patients had antitumor activity not previously reported, all with fusion tyrosine kinases or validated leukemia targets: a patient younger than 5 years with a *MET* fusion–positive malignant supratentorial tumor treated with the *MET* inhibitor capmatinib; a patient younger than 5 years with a *FGFR1* fusion brain stem high-grade glioma treated with the multitargeted tyrosine kinase inhibitor (MTKI) covering *FGFR1* lenvatinib; a patient aged between 5 and 10 years with Burkitt lymphoma treated with an anti-CD20/CD3 antibody.

According to the predefined criteria for activity assessment, there were 5 cohorts with 10 or more patients treated by the same medicine for the same indication. Three cohorts of patients were treated with the *MEK* inhibitor trametinib: cohort 1 (*BRAF *fusion–positive LGG), cohort 2 (*NF1*-related optic pathway glioma), and cohort 3 (*NF1*-related plexiform neurofibroma). Patients in cohort 4 (*BRAF*-altered LGG) were treated by the combination of *BRAF*/*MEK* inhibitors (dabrafenib and trametinib), and patients in cohort 5 (osteosarcoma) were treated with the MTKI regorafenib. Patient characteristics and reported outcomes are detailed in Table 3.

**Table 3.** Patient Characteristics, Reported Outcomes, and Adverse Drug Reactions of the 5 Cohorts of ≥10 Patients Treated With the Same Medicine and Indication

Cohort No.	Innovative therapy	Tumor type	Biomarker	Patients, No.	Age at inclusion, median (range), y	Prior lines of therapy, median (range)	Time since diagnosis, median (range), y	ORR/PFS	PFS, median (range), d	Patients still on therapy at last follow-up	SACHA recommendation to SFCE centers
1	Trametinib	Low grade glioma	*BRAF* fusion	23	6.8 (0.7-16.5)	3 (1-10)	4.1 (0.4-14.8)	ORR, 5/23 (5 PR)	275 (40-663)	12/23	Continue
2	Trametinib	Low grade glioma	*NF1* germline variant	13	7.6 (3.3-13.7)	2 (1-5)	4.4 (2.0-10.4)	ORR, 4/13 (4 PR)	334 (222-576)	6/13	Continue	
3	Trametinib	Plexiform neurofibroma	*NF1* germline variant	10	6.5 (0.3-13.5)	0 (0-1)	2.7 (0.1-7.1)	ORR, 0/10	455 (103-786)	9/10	No longer prescription	
4	Dabrafenib /trametinib	Low grade glioma	*BRAF* variant	28	10.5 (0.8-19.2)	2 (0-9)	3.5 (0.2-16.5)	ORR, 19/27 (18 PR, 1 CR)	372 (34-796)	21/28	Continue	
5	Regorafenib	Osteosarcoma	None	13^a^	17.3 (10.9-22.8)	2 (1-6)	1.8 (0.7-13.6)	4-mo PFS, 37%	125 (26-513)	5/13	Continue	

Abbreviations: CR, complete response; ORR, objective tumor responses; PFS, progression-free survival; PR, partial response; SACHA, Securing Access to Innovative Therapies for Children, Adolescents, and Young Adults with Cancer Used Outside Clinical Trials; SFCE, French Society of Pediatric Oncology.

^a^ Two patients not evaluable (follow-up <4 months, and regorafenib ongoing at last follow-up).

## Discussion

The SACHA-France study is a unique innovative initiative that collects clinical data on safety and activity of compassionate or off-label new anticancer medicines prescribed outside clinical trials for children, adolescents, and young adults. It is worth nothing that all French pediatric hematology-oncology centers are actively involved, with more than 150 patients included annually and 55 different drugs prescribed.

The goal of SACHA is not to encourage treating patients with innovative medicines outside clinical trials. For this reason, multidisciplinary discussion has been set up and is mandatory to ensure that all relevant options, starting with enrollment in a clinical trial, are identified and discussed for each patient at the time of disease recurrence. Nevertheless, compassionate and off-label prescriptions do happen and SACHA is collecting this information in a real-life setting, to our knowledge for the first time, to assure patient security and to inform antitumoral activity and toxicity.

Off-label and compassionate use prescription is common in pediatric oncology and its prevalence has increased over the last decade; however, only limited data are published and safety information remains largely underreported.^5^ This increasing prevalence was confirmed in a retrospective single-institution cohort with 374 patients who received an off-label targeted anticancer drug from 2007 to 2017, with encouraging activity data, but with clinically significant ADRs (38%) and discontinuation due to toxic effects (13%).^6^ In a 5-year period retrospective review of all genomically targeted therapeutic single patient use in 4 US large pediatric cancer centers, ORRs were seen in 39.5% cases, treatment discontinuation due to toxicity was reported in 13% patients, but no information on other ADRs were reported, strongly reinforcing the high need of systematically and adequate collection of this data at the multi-center level.^7^

In SACHA-France, there was a 25% reported ORR in patients with solid tumors, brain tumors, and lymphomas mostly related to innovative drugs already explored within pediatric clinical trials for either the same disease or biomarker match but that are not yet approved in children and adolescents. This is for example the case of the combination of dabrafenib with trametinib in pediatric patients with *BRAF* V600E variant LGG.^4,8^ In March 2023, this combination granted FDA approval for patients ages 1 year and older with LGG with a *BRAF* V600E variation who require systemic therapy, but is not yet approved by the EMA. Therefore, the only access in Europe to this proven effective combination for children and adolescents continues to be off-label (tablets) or compassionate (oral formulation) use. Similarly, *MEK* inhibitors have been extensively explored in pediatric patients with *BRAF* fusion positive or *NF1*-related tumors. Selumetinib has shown promising single-agent activity in this group of patients^9^ and has been approved since October 2020 by the FDA and June 2021 by the EMA for the treatment of inoperable symptomatic plexiform neurofibromas (PNs) in children with *NF1*. Additionally, trametinib has shown single-agent activity in this population,^10^ but has not been approved for this last indication by the FDA or the EMA. Due to initial lack of access to selumetinib until EMA approval in June 2021, trametinib was prescribed for this indication in Europe until that date.

In our study, we confirmed previously reported ORR within early phase clinical trials, although in our study ORR of *BRAF*-variant LGG to dabrafenib plus trametinib was higher that previously reported,^4^ while similar for patients with *BRAF* fusion–positive LGG and *NF1*-related optic pathway gliomas treated with trametinib.^11^ These differences have to be interpreted with caution due to the lack of independent radiological review, which has been shown to significantly modify the final response assessment for these tumors.^12^ In our study, none of the 10 patients with *NF1*-related PNs treated by trametinib had an ORR, compared with 24% ORR reported,^11^ but most had a prolonged clinical benefit. This difference may reflect a more selected population in clinical trials, compared with a real-life prescription population. Based on these results and current selumetinib EMA approval for this indication, the SACHA steering committee recommended to no longer prescribe trametinib, and instead prescribe selumetinib for patients with *NF1*-related inoperable PNs. MTKIs with antiangiogenic activities have also shown activity in patients with bone sarcomas. Regorafenib is a MTKI with proven efficacy in adults with osteosarcoma in 2 randomized double-masked trials^13,14^ but with limited experience in pediatric patients.^15^ In our study, regorafenib 4-month PFS in patients with osteosarcoma was similar to other single-agent MTKI reported.^16^ Currently MTKIs are being included in upfront trials in combination with chemotherapy (NCT05691478, NCT05830084) and as maintenance therapy (NCT04055220, NCT04698785).

The SACHA-France study allowed the early identification of exceptional responders to inform further pediatric development of those drugs within clinical trials. This is the case of the antitumor activity not previously reported in patients with *MET* and *FGFR1* fusion–positive CNS tumors. The first case supported the development of the capmatinib plus everolimus arm in the ESMART trial (NCT02813135). This basket trial already has an arm with a FGFR inhibitor (futibatinib), which opened to enrollment after that patient had already been treated by lenvatinib. This underscores the role of the SACHA-France study in supporting the formal development of drugs in the pediatric population when antitumor activity is observed and not as a substitute for them. Although quality of data derived from off-label and compassionate use is not equivalent to clinical trial data, clinical data can support clinical trial data in the regulatory review of new drug applications or indications, and they increasingly are used in regulatory decision making.^17,18^ Nevertheless, in very rare populations with strong predictive biomarkers this may even lead directly to regulatory approval, as was the case of alpelisib, which obtained FDA accelerated approval for adult and pediatric patients with severe manifestations of *PIK3CA*-related overgrowth spectrum.^19^

The SACHA-France study has an important pharmacovigilance objective with remote monitoring of all reported ADRs and special focus on unexpected and SADRs. This allows improving the knowledge of clinical toxic effects to innovative therapies that are otherwise usually underreported.^20^ Since March 2020, almost 300 ADRs have been reviewed and 24 SADRs reported to the national competent authorities. This includes, for example, a secondary malignant neoplasm in a patient younger than 5 years with ATRT and a long-lasting response to tazemetostat, which is the second case reported to our knowledge.^21^ Interestingly, some of the reported unexpected SADRs are well-known toxic effects already reported to a specific drug or drug class but not included in the summary of product characteristics (eg, alectinib and psychiatric ADRs^22^; regorafenib and pneumothorax^23^), further stressing the importance of adequate reporting of these toxic effects.

It is important to highlight the strong support to this study by ANSM, the French competent authority responsible for approving the compassionate access of unauthorized drugs that represents half of the drugs prescribed in our study. In France, early drug access before marketing authorization is granted by ANSM through the Temporary Authorization for Use program (since July 2021 called early access and compassionate access programs) that allows patients with unmet medical needs to be treated with an innovative drug before marketing authorization, if the assessment of preliminary data presents a positive risk-benefit balance and the patient has no therapeutic alternatives among the existing therapies and clinical trials.^24^ Since 2021, ANSM actively recommends the inclusion of pediatric patients treated on a compassionate use program in the SACHA-France study in order to ensure adequate monitoring of these prescriptions. In 2017, the EU Commission Expert Group on Safe and Timely Access to Medicines for Patients (STAMP) released the results of a study of the public health aspects related to the off-label use of medicinal products, investigating the balance between the benefits and risks for patients, and the regulatory framework for the off-label use of medicines.^25^ Following the proposal by the STAMP Group, a medicines repurposing framework is being implemented in the EU.^26^ In this context the SACHA initiative could provide, beyond pediatric oncology, relevant information from real patient populations while assuring safety of patients receiving off-label treatments. Based on the SACHA-France experience, the ITCC has developed the SACHA International Project (ITCC-105) that will be launched in 2023.

### Limitations

The SACHA-France study is limited by the absence of information on off-label prescriptions in France to calculate the proportion of patients enrolled. This information will be available in the near future for patients within compassionate use programs thanks to the collaboration with the ANSM.

## Conclusions

In this cohort study, we confirmed the feasibility of clinical safety and activity data collection of compassionate and off-label new anticancer medicines prescribed to children, adolescents, and young adults. Our study allowed the adequate pharmacovigilance reporting of unexpected and serious ADRs, confirmation of antitumor responses in real-life situation and the early identification of exceptional responses allowing further pediatric drug development within clinical trials.

## Notes

Supplement 1.
eMethods.
**eTable 1.** SACHA Screening Failures **eTable 3.** Characteristics of All Nonserious Adverse Drug Reactions (ADRs) Reported in the SACHA France Study

Supplement 2. **eTable ****2.** Description of Characteristics of Patients and Prescriptions **eTable 4.** Characteristics of Patients With Solid Tumors, Brain Tumors, or Lymphomas With Objective Responses Reported

Supplement 3.
Data Sharing Statement
