# Supplementary material for: Measuring Safety and Outcomes for the Use of Compassionate and Off-Label Therapies for Children, Adolescents, and Young Adults With Cancer in the SACHA-France Study
Source: JAMA Netw Open. 2023 Jul 3;6(7):e2321568. doi: 10.1001/jamanetworkopen.2023.21568 (PMC10318477; doi:10.1001/jamanetworkopen.2023.21568)
Supplement: Supplement 1. — eMethods. eTable 1. SACHA Screening Failures eTable 3. Characteristics of All Nonserious Adverse Drug Reactions (ADRs) Reported in the SACHA France Study [file jamanetwopen-e2321568-s001.pdf]

Supplemental Online Content

Berlanga P, Ndounga-Diakou LA, Aerts I, et al. Measuring safety and outcomes for the use of compassionate and off-label therapies for children, adolescents, and young adults with cancer in the SACHA-France study. *JAMA Netw Open.* 2023;6(7):e2321568.
doi:10.1001/jamanetworkopen.2023.21568

eMethods.

eTable 1. SACHA Screening Failures

eTable 3. Characteristics of All Nonserious Adverse Drug Reactions (ADRs) Reported in the SACHA France Study

This supplemental material has been provided by the authors to give readers additional information about their work.

© 2023 Berlanga P et al. *JAMA Network Open.*

eMethods

In France, patients < 18 years are treated in one of the 30 SFCE centers, which constitute a national network divided in 7 Interregional Pediatric Oncology Networks (OIR) where all new patients are discussed in Interregional Pediatric multidisciplinary tumor boards (RCPPI). Before inclusion in the SACHA-France study, RCPPI discussion is mandatory to ensure that all patients receive the most adapted available treatment, preferably within clinical trials. In the absence of an available trial, or when the patient is not eligible or enrolment refused by patients/parents, the best possible therapeutic option available is proposed and the possibility of inclusion in the SACHA-France study if the patient fulfils the inclusion criteria.

Information collected in the SACHA study include: molecular details supporting the choice of the innovative treatment (if indicated), type of cancer, stage and lines of previous treatments as well as innovative treatment dosage, start date, dose reductions, temporary or permanent discontinuation and causes of change in dosage or treatment stop. Follow-up information is collected every 3 months until end of treatment or patient's death documenting tumor response and adverse drug reactions (ADRs).

VIGINOM is a proactive system for monitoring adverse new drug reactions in oncology. It has been set up by the UFPV of Gustave Rousy and validated in the field of pharmacoepidemiology. This secure web portal was initially designed to better characterize the adverse effects of new drugs, under real-life conditions, from the early access/compassionate use phase and then during the first year after marketing authorization. It allows the automatic generation and sending of regulatory pharmacovigilance forms to the Regional Pharmacovigilance Centers and to the marketing authorisation holders. Within the framework of the French SACHA study, VIGINOM has been improved to allow the collection of clinical, molecular and biological data from patients.

Progression-free survival (PFS) and best response from the start of treatment to tumor relapse/progression is assessed by the patient's treating physician, following the standard response criteria for each tumor type. Patients who clinically deteriorated before formal assessment are considered as progressive. For all patients with solid/brain tumors and lymphomas and measurable/evaluable disease at study entry and reported objective response (partial response (PR)/ complete response (CR)), radiological reports at baseline/time of response are reviewed by the SACHA-France coordinating investigator to confirm the coherence of the reported response.

The SACHA France Steering Committee is composed of the SACHA France coordinating investigator, one pediatric hemato-oncologist representative from each of the 7 OIRs, the UFPV of Gustave Roussy and a representative from the sponsor. The SACHA France Steering Committee meets every two months to steer the implementation of the project, control the overall quality and review main safety and activity data.

© 2023 Berlanga P et al. *JAMA Network Open.*

e Table 1. SACHA Screening Failures (n=15)

Patient	Reason screening failure
1	Marketing authorization before 2007 (bevacizumab)
2	Marketing authorization before 2007 (bevacizumab)
3	Marketing authorization before 2007 (bevacizumab)
4	Marketing authorization before 2007 (bevacizumab)
5	Marketing authorization before 2007 (sirolimus)
6	On-label use (denosumab, giant cell tumor of the bone, 13 years old)
7	Patient > 25 years old (trametinib, low-grade glioma)
8	On-label use (brentuximab/nivolumab, Hodgkin lymphoma, 19 years old)
9	Duplicate patient
10	Marketing authorization before 2007 (sirolimus)
11	Patient > 25 years old (trametinib, low-grade glioma)
12	On-label use (denosumab, giant cell tumor of the bone, 16 years old)
13	On-label use (pembrolizumab, Hodgkin lymphoma, 18 years old)
14	On-label use (blinatumomab, B-Acute lymphoblastic leukemia, 7 years old)
15	On-label use (blinatumomab, B-Acute lymphoblastic leukemia, 10 years old)

© 2023 Berlanga P et al. *JAMA Network Open.*

eTable 3. Characteristics of All Nonserious Adverse Drug Reactions (ADRs) Reported in the SACHA France Study

	All grade N(%)	Grade 2 N(%)	Grade 3 N(%)	Grade 4 N(%)
Number of patients who received at least one dose of treatment	351			
Number of patients with at least one ADR	121 (34.4)*	94 (26.7)**	53 (15.0)***	5 (1.4)****
Blood and lymphatic system disorders				
Anemia	2 (0.5)	NA	2 (0.5)	0(0)
Febrile neutropenia	2 (0.5)	0(0)	2 (0.5)	0(0)
Eye disorders				
Keratitis	2 (0.5)	2 (0.5)	0(0)	0(0)
Gastrointestinal disorders				
Abdominal pain	3 (0.8)	3 (0.8)	0(0)	0(0)
Constipation	3 (0.8)	3 (0.8)	0(0)	0(0)
Diarrhea	16 (4.5)	14 (3.9)	3 (0.8)	0(0)
Dysphagia	1 (0.2)	2 (0.5)	0(0)	0(0)
Mucositis oral	12 (3.4)	11 (3.1)	1 (0.2)	0(0)
Nausea	6 (1.7)	6 (1.7)	0(0)	0(0)
Pancreatitis	1 (0.2)	2 (0.5)	0(0)	0(0)
Vomiting	10 (2.8)	9 (2.5)	1 (0.2)	0(0)
General disorders and administration site conditions				
Canker sore				
Abdominal pain	1 (0.2)	1 (0.2)	0(0)	0(0)
Asthenia	13 (3.7)	9 (2.5)	4 (1.1)	0(0)
Fever	8 (2.2)	6 (1.7)	2 (0.5)	0(0)
Pain	4 (1.1)	2 (0.5)	3 (0.8)	0(0)
Infections and infestations				
Bacteremia	1 (0.2)	0(0)	1 (0.2)	0(0)
Eye infection	1 (0.2)	1 (0.2)	0(0)	0(0)
Folliculitis	1 (0.2)	0(0)	1 (0.2)	0(0)
Paronychia	20 (5.6)	14 (3.9)	6 (1.7)	

© 2023 Berlanga P et al. *JAMA Network Open.*

	All grade N(%)	Grade 2 N(%)	Grade 3 N(%)	Grade 4 N(%)
Number of patients who received at least one dose of treatment	351			
Number of patients with at least one ADR	121 (34.4)*	94 (26.7)**	53 (15.0)***	5 (1.4)****
Infections and infestations				
Sepsis	1 (0.2)	0(0)	1 (0.2)	0(0)
Whitlow	2 (0.5)	1 (0.2)	1 (0.2)	0(0)
Pyelonephritis	1 (0.2)	0(0)	1 (0.2)	0(0)
Injury, poisoning and procedural complications				
Fracture	1 (0.2)	1 (0.2)	0(0)	0(0)
Investigations				
Alanine aminotransferase increased	5 (1.4)	0(0)	5 (1.4)	0(0)
Aspartate aminotransferase increased	2 (0.5)	NA	2 (0.5)	0(0)
CPK increased	3 (0.8)	NA	1 (0.2)	2 (0.5)
Neutrophil count decreased	8 (2.2)	0(0)	6 (1.7)	2 (0.5)
Platelet count decreased	2 (0.5)	0(0)	1 (0.2)	1 (0.2)
Weight gain	4 (1.1)	3 (0.8)	1 (0.2)	0(0)
Weight loss	2 (0.5)	2 (0.5)	0(0)	0(0)
Metabolism and nutrition disorders				
Hyponatremia	1 (0.2)	0(0)	1 (0.2)	0(0)
Anorexia	2 (0.5)	2 (0.5)	0(0)	0(0)
Renal and urinary disorders				
Hematuria	1 (0.2)	0(0)	1 (0.2)	0(0)
**Respiratory, thoracic and mediastinal disorders**				
Pneumothorax	2 (0.5)	2 (0.5)	0(0)	0(0)
Skin and subcutaneous tissue disorders				
Acneiforme dermatose	1 (0.2)	1 (0.2)	0(0)	0(0)
Dermatitis	3 (0.8)	2 (0.5)	0(0)	1 (0.2)
Dry skin	8 (2.2)	5 (1.4)	3 (0.8)	0(0)

© 2023 Berlanga P et al. *JAMA Network Open.*

	All grade N(%)	Grade 2 N(%)	Grade 3 N(%)	Grade 4 N(%)
Number of patients who received at least one dose of treatment	351			
Number of patients with at least one ADR	121 (34.4)*	94 (26.7)**	53 (15.0)***	5 (1.4)****
Skin and subcutaneous tissue disorders				
Eczema	7 (1.9)	3 (0.8)	4 (1.1)	0(0)
Erythema	2 (0.5)	2 (0.5)	0(0)	0(0)
Folliculitis	2 (0.5)	1 (0.2)	1 (0.2)	0(0)
Impetigo	1 (0.2)	2 (0.5)	0(0)	0(0)
Intertrigo	1 (0.2)	2 (0.5)	0(0)	0(0)
Pain of skin	1 (0.2)	1 (0.2)	0(0)	0(0)
Palmar-plantar erythrodysesthesia syndrome	6 (1.7)	3 (0.8)	3 (0.8)	0(0)
Rash acneiform	10 (2.8)	9 (2.5)	1 (0.2)	0(0)
Rash maculo-papular	8 (2.2)	4 (1.1)	4 (1.1)	0(0)
canker sore	1 (0.2)	1 (0.2)	0(0)	0(0)
Skin mycositis	1 (0.2)	1 (0.2)	0(0)	0(0)
Skin ulceration	1 (0.2)	1 (0.2)	0(0)	0(0)
Pruritis	2 (0.5)	2 (0.5)	0(0)	0(0)
Xerosis	1 (0.2)	0(0)	1 (0.2)	0(0)
Vascular disorders				
Hypertension	2 (0.5)	1 (0.2)	1 (0.2)	0(0)
Musculoskeletal and connective tissue disorders				
Bone pain	1 (0.2)	0(0)	0(0)	0(0)
Back pain	1 (0.2)	1 (0.2)	0(0)	0(0)
Arthralgia	2 (0.5)	0(0)	2 (0.5)	0(0)
Ear and labyrinth disorders				
Otitis	1 (0.2)	1 (0.2)	0(0)	0(0)

*Two patients with unknown ADR's grade (1- Thyroid-stimulating hormone increased; 2-Aspartate aminotransferase increased). ** Forty patients with more than one ADRs; *** Fourteen patients with at least two ADRs; **** One patient with two ADRs.

© 2023 Berlanga P et al. *JAMA Network Open.*
