# Supplementary material for: Measuring Safety and Outcomes for the Use of Compassionate and Off-Label Therapies for Children, Adolescents, and Young Adults With Cancer in the SACHA-France Study
Source: JAMA Netw Open. 2023 Jul 3;6(7):e2321568. doi: 10.1001/jamanetworkopen.2023.21568 (PMC10318477; doi:10.1001/jamanetworkopen.2023.21568)
Supplement: Supplement 3. — Data Sharing Statement [file jamanetwopen-e2321568-s003.pdf]

Data Sharing Statement

Berlanga. Measuring Safety and Outcomes for the Use of Compassionate and Off-Label Therapies for Children, Adolescents, and Young Adults With Cancer in the SACHA-France Study. *JAMA Netw Open*. Published July 03, 2023. doi:10.1001/jamanetworkopen.2023.21568

Data
Data available: Yes
Data types: Deidentified participant data
How to access data: pablo.berlanga@gustaveroussy.fr
When available: With publication

Supporting Documents
Document types: None

Additional Information
Who can access the data: Researchers whose proposed use of the data has been approved
Types of analyses: For academic purposes only
Mechanisms of data availability: After approval of a proposal
